# Safety and Effectiveness of Low‐Density Lipoprotein Cholesterol–Lowering Therapy With Evolocumab for Familial Hypercholesterolemia/Hypercholesterolemia in Japan: A Real‐World, Postmarketing, Single‐Arm Study

**DOI:** 10.1161/JAHA.124.035809

**Published:** 2024-10-29

**Authors:** Koutaro Yokote, Junya Ako, Kazuo Kitagawa, Nobuhiro Osada, Feng Sheng, Masae Sonoda, Tamio Teramoto

**Affiliations:** ^1^ Department of Endocrinology, Hematology and Gerontology Chiba University Graduate School of Medicine Chiba Japan; ^2^ Department of Cardiovascular Medicine Kitasato University School of Medicine Sagamihara Japan; ^3^ Department of Neurology Tokyo Women’s Medical University School of Medicine Tokyo Japan; ^4^ Medical Affairs Amgen K.K. Tokyo Japan; ^5^ Global Patient Safety Amgen K.K. Tokyo Japan; ^6^ Teikyo Academic Research Center Teikyo University Tokyo Japan

**Keywords:** adverse event, evolocumab, familial hypercholesterolemia, hypercholesterolemia, LDL‐C–lowering therapy, Lipids and Cholesterol

## Abstract

**Background:**

Evolocumab is the first monoclonal antibody against proprotein convertase subtilisin/kexin type 9 approved in Japan for familial hypercholesterolemia (FH) and hypercholesterolemia; however, data on its safety and effectiveness in the real‐world setting in Japan are limited.

**Methods and Results:**

This real‐world, postmarketing, single‐arm study assessed the safety and effectiveness of low‐density lipoprotein cholesterol lowering with evolocumab in patients with homozygous/heterozygous familial hypercholesterolemia and hypercholesterolemia with high risk in Japan (668 sites). The primary safety end point was the incidence (percentage) and number of patients with adverse drug reactions and serious adverse events during the 104‐week follow‐up. Primary effectiveness end points included the percentage change in low‐density lipoprotein cholesterol levels from baseline to week 12, assessed using 2‐sided paired *t* tests. The safety and effectiveness sets comprised 3724 (homozygous FH, n=108; heterozygous FH, n=2009; hypercholesterolemia with high risk, n=1607) and 2797 (homozygous FH, n=91; heterozygous FH, n=1615; hypercholesterolemia with high risk, n=1091) patients, respectively. Overall, mean age and disease duration were 63.2 and 12.3 years, respectively. Serious adverse drug reactions and serious adverse events were experienced by 0.5% and 10.3% of patients; the incidence rates of myocardial infarction and stroke were 0.7% and 0.3%, respectively. A significant mean±SD percentage change in low‐density lipoprotein cholesterol levels was observed at week 12 among patients with homozygous FH (−45.7%±28.2; *P*<0.001), heterozygous FH (−55.9%±28.8; *P*<0.001), and hypercholesterolemia with high risk (−63.3%±23.7; *P*<0.001).

**Conclusions:**

Evolocumab was well tolerated, and real‐world patients with familial hypercholesterolemia and hypercholesterolemia with high risk in Japan had sustained low‐density lipoprotein cholesterol reduction.

**REGISTRATION:**

URL: https://www.clinicaltrials.gov; Unique Identifier: NCT02808403.

**Nonstandard Abbreviations and Acronyms**
ADR: adverse drug reaction
AE: adverse event
CVD: cardiovascular disease
EXPLORE‐J: Examination of Patient Outcomes in Real‐World Practice Settings in Japan
FH: familial hypercholesterolemia
FOURIER: Further Cardiovascular Outcomes Research With PCSK9 Inhibition in Subjects With Elevated Risk
FOURIER‐OLE: Further Cardiovascular Outcomes Research With PCSK9 Inhibition in Subjects With Elevated‐Risk–Open Label Extension
GOULD: Getting to an Improved Understanding of Low‐Density Lipoprotein‐Cholesterol and Dyslipidemia Management
HeFH: heterozygous familial hypercholesterolemia
HoFH: homozygous familial hypercholesterolemia
HEYMANS: cHaractEristics of hYperlipidaeMic pAtieNts at initiation of evolocumab and treatment patternS
OSLER: Open‐Label Study of Long‐Term Evaluation against LDL Cholesterol
PCSK9: proprotein convertase subtilisin/kexin type 9
SAE: serious adverse event

**Clinical Perspective**

**What is New?**

- Evolocumab was safe and effective in Japanese patients with homozygous/heterozygous familial hypercholesterolemia and hypercholesteremia with high risk in real‐world practice.
- Low incidence rates of serious adverse events (10.3%) and serious adverse drug reactions (0.5%) and reductions in low‐density lipoprotein cholesterol levels by 49.6% (homozygous familial hypercholesterolemia), 54.4% (heterozygous familial hypercholesterolemia), and 60.1% (hypercholesterolemia with high risk) were observed at 104 weeks with evolocumab.
- The predominant proportion of patients initiating evolocumab shifted from those with heterozygous familial hypercholesterolemia in 2016 to those with hypercholesterolemia with high risk in 2020.

**What Are the Clinical Implications?**

- This study provides evidence for the favorable benefit–risk balance of evolocumab in patients with familial hypercholesterolemia and hypercholesterolemia with high risk in the Japanese population.
- This study helps to provide a good solution to achieve target low‐density lipoprotein cholesterol levels in Asian patients who cannot continue statin treatment due to safety problems.

Cardiovascular disease (CVD) is the leading cause of death worldwide, accounting for >30% of the total global disease burden among all major diseases, injuries, and risk factors that affect the mortality rate.^1^ Elevated low‐density lipoprotein cholesterol (LDL‐C) is a major modifiable risk factor for CVD development and a validated surrogate end point for cardiovascular risk reduction.^2^ Interventional studies, epidemiological studies, and meta‐analyses have shown that LDL‐C is a strong independent predictor of coronary heart disease risk across diverse patient groups, including Japanese populations.^3^, ^4^, ^5^, ^6^, ^7^ Studies of both gain‐ and loss‐of‐function genetic variants provide additional evidence supporting LDL‐C as a therapeutic target and a surrogate for cardiovascular outcomes.^8^, ^9^

The Japan Atherosclerosis Society guidelines recommend a target LDL‐C goal of <70 mg/dL for secondary prevention in patients with high‐risk conditions such as familial hypercholesterolemia (FH) and history of acute coronary syndrome.^10^ On the other hand, the European Society of Cardiology/American College of Cardiology guidelines suggest a more aggressive LDL‐C goal of <55 mg/dL.^11^, ^12^ Despite the widespread use of lipid‐lowering therapies such as statins, target LDL‐C goals are often not achieved, which could be attributed to several factors, including the dose of statins used, frequency of uptitration, and compliance. Indeed, in the EXPLORE‐J (Examination of Patient Outcomes in Real‐World Practice Settings in Japan) registry investigation of hospitalized patients with acute coronary syndrome, 32.2% were treated with lipid‐lowering therapies (intensive statin therapy, 5.8%) at hospitalization, and only 14.4% had an LDL‐C level of <70 mg/dL within 14 days of hospitalization.^13^

Evolocumab is a fully human monoclonal immunoglobulin G2 that selectively binds with high affinity to circulating PCSK9 (proprotein convertase subtilisin/kexin type 9) to inhibit the PCSK9–LDL receptor interaction, thereby preventing PCSK9‐mediated LDL receptor degradation and increasing LDL‐C reduction.^14^ Evolocumab is the first monoclonal antibody approved in Japan for FH and hypercholesterolemia.^15^ The FOURIER (Further Cardiovascular Outcomes Research With PCSK9 Inhibition in Subjects With Elevated Risk) study previously assessed evolocumab in a population of patients with atherosclerotic CVD (eg, a history of myocardial infarction, nonhemorrhagic stroke, and symptomatic peripheral artery disease). This primary study demonstrated that evolocumab effectively reduced LDL‐C by 59% and reduced the risk of cardiovascular events, with a similar rate of adverse events (AEs), compared with a placebo group who received standard therapy, over a median follow‐up period of 2.2 years.^16^ Furthermore, a pooled analysis of Japanese patients with elevated LDL‐C and high cardiovascular risk participating in the OSLER‐1 (Open‐Label Study of Long‐Term Evaluation against LDL Cholesterol) and OSLER‐2 studies revealed that evolocumab was well tolerated in this population and demonstrated a sustained reduction in LDL‐C at 12 months.^17^, ^18^, ^19^

This survey‐based surveillance study investigated the real‐world use of evolocumab in Japanese patients as part of the postmarketing pharmacovigilance‐focused investigations documented in the Japan Risk Management Plan. This study aimed to determine the real‐world use and effectiveness of evolocumab, as well as the occurrence of AEs and adverse drug reactions (ADRs) in patients with homozygous FH (HoFH), heterozygous FH (HeFH), and hypercholesterolemia with high risk over 104 weeks of follow‐up in Japan.

## Methods

### Data Availability

Qualified researchers may request data for Amgen clinical studies. Complete details are available at the following link: https://wwwext.amgen.com/science/clinical‐trials/clinical‐data‐transparency‐practices/.

#### Study Design

This observational, postmarketing, single‐arm study prospectively enrolled (June 24, 2016, to October 31, 2020) real‐world Japanese patients treated with evolocumab for HoFH, HeFH, or hypercholesterolemia with a high risk of cardiovascular events (history of coronary artery disease [CAD], noncardiogenic cerebral infarction, peripheral artery disease, diabetes, chronic kidney disease) and statin intolerance (NCT02808403). The safety and effectiveness of evolocumab were assessed using separate patient analysis groups. The index date was defined as the date of the first evolocumab injection administered to the patient. Demographic and clinical data were collected at enrollment. The baseline period was defined as 8 weeks before the index date. Patients were followed up for 104 weeks until death, loss to follow‐up, or a 30‐day gap since the last evolocumab injection. No stopping rules were applied.

#### Study Population and Setting

This study was conducted at 668 clinical sites in Japan between June 24, 2016, and April 20, 2023. Eligible patients were required to have a diagnosis of HoFH, HeFH, or hypercholesterolemia with a high risk of cardiovascular events and an insufficient response to a 3‐hydroxy‐3‐methylglutaryl coenzyme A reductase inhibitor (statin) treatment. An insufficient response to statin treatment includes patients who cannot take any statin due to statin intolerance. Off‐label use data were collected. The enrolled patients mainly complied with the efficacy and dosage regimen within the Japanese evolocumab package insert. Patients eligible for the safety analysis had at least 1 documented visit after the index date. Patients in the safety analysis group were excluded only if there was a breach of contract or violation of registration. Patients in whom follow‐up LDL‐C data were available and who received concomitant statin were included in the effectiveness analysis. Effectiveness analysis group 1 included patients who were regarded as evaluable for efficacy analysis of evolocumab. Effectiveness analysis group 1 excluded patients with evolocumab use without statins during the 104‐week observation period, those with inadequate lipid or LDL‐C data, those with no recorded visits after the first administration date, and those judged inappropriate for effectiveness analysis. Statin‐intolerant patients were included in effectiveness analysis group 1 as it was on‐label use; however, patients who were not receiving concomitant statins without a diagnosis of statin intolerance were excluded from this group. Effectiveness analysis group 2 included only patients with on‐label use of evolocumab (complying with the package insert) and excluded those not on statin therapy at the time of evolocumab initiation or exceeding the specified dose to analyze on‐label use only, those with other contraindications, and those aged <15 years.

#### Data Collection and Safety Reporting Measures

All patient data were collected in a patient registration form or a case report form using the Electronic Data Capture system (PostMaNet, Fujitsu, Tokyo). The following data were collected at baseline: sex, age, body mass index, inpatient/outpatient setting at the time of evolocumab initiation, diagnosis (HoFH/HeFH/hypercholesterolemia), and past medical history, including CAD, diabetes/glucose intolerance, chronic kidney disease, noncardiogenic cerebral infarction, peripheral artery disease, hypertension, low high‐density lipoprotein cholesterol (defined as <40 mg/dL), smoking history, family history of premature CAD, statin use intensity (low, moderate, high), concomitant lipid‐lowering medications, and prior medications. Data management was conducted by an external service provider (A2 Healthcare Corporation, Tokyo, Japan) under the supervision of Amgen K.K.

#### Definitions

AEs were defined as any untoward medical occurrence in a patient, irrespective of the causal relationship with evolocumab, including abnormal laboratory or anti–evolocumab antibody findings, symptoms or disease temporally associated with evolocumab, worsening of a preexisting condition or an underlying disease, or those associated with discontinuation. A serious adverse event (SAE) was defined as any AE that caused fatality, was life‐threatening, required overnight hospitalization or prolongation of existing hospitalization, resulted in a persistent or significant disability/inability, was a congenital anomaly/birth defect, or was any other significant medical hazard. ADRs were defined as AEs reported by physicians other than those assessed as “not related” by physicians (including unknown/not specified). The incidence rates of myocardial infarction and stroke were assessed by the Food and Drug Administration custom query.^20^

#### Study Objectives and End Points

The objective of this study was to determine the safety and effectiveness of evolocumab for up to 104 weeks after initiation. The primary safety end points were the incidence (percentage) and number of patients with ADRs and SAEs during the 104‐week follow‐up period. The primary effectiveness end point was the percentage change in LDL‐C levels from baseline to 12 weeks after evolocumab initiation.

Other safety end points included the incidence (percentage), number of patients, and number of AEs and ADRs per 1000 person‐years during the 104‐week follow‐up period. Secondary effectiveness end points included the percentage change in LDL‐C from baseline to 4, 12, 24, 52, 76, and 104 weeks and percentage change in non–high‐density lipoprotein cholesterol, total cholesterol, and triglycerides from baseline to 4, 12, 24, 52, 76, and 104 weeks.

#### Ethics Statement

The study was approved by the institutional or ethics review board at each participating site or by a central ethical review board (Public Health Research Foundation, Tokyo). The study was conducted in accordance with the Pharmaceutical and Medical Devices Law, Good Post‐Marketing Study Practice in Japan, and the Declaration of Helsinki. The study sponsors take responsibility for the accuracy and completeness of the data and analyses. Written informed consent was provided by all enrolled patients.

#### Statistical Analysis

A target sample size of 3300 patients with HoFH, HeFH, or hypercholesterolemia with high risk was selected to provide 95% statistical power for safety analysis, with an assumed discontinuation rate of 10% over 104 weeks. This allowed for AEs with an incidence of 0.1% to be detected in at least 1 patient with a probability of 95% during the observation period of 104 weeks. Categorical variables were reported as frequencies and percentages. Continuous variables were reported as mean±SD or median (first quartile to third quartile). Confidence intervals (95%) were calculated. Two‐sided paired *t* tests were used to assess if there was a significant difference between LDL‐C levels at baseline and at week 12. Statistical analyses were conducted by an external vendor (A2 Healthcare Corp, Tokyo, Japan) in accordance with the statistical analysis plan approved by Amgen K.K. (Medical Dictionary for Regulatory Activities version 26.0).

## Results

### Demographic and Clinical Characteristics of the Safety Analysis Group

**Figure 1:**
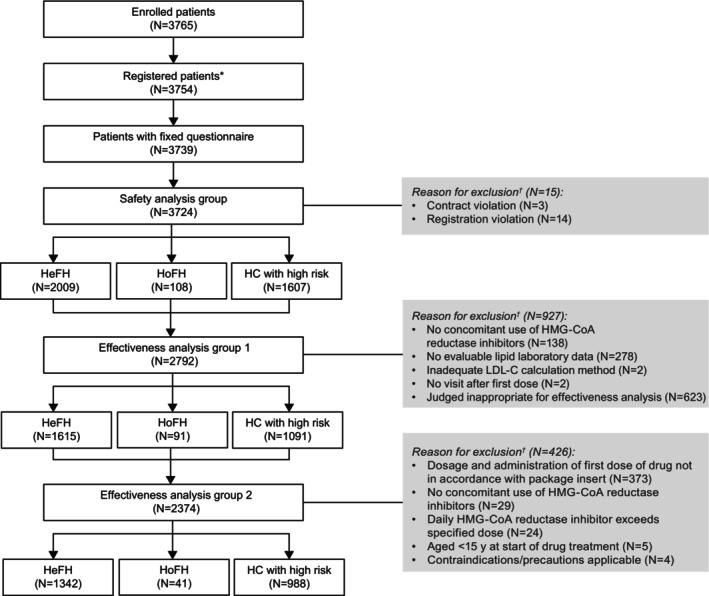
Patient disposition flowchart and attrition. *When multiple reasons for exclusion existed, all relevant reasons were included in the tabulation. ^†^Statin‐intolerant cases were not eligible for this exclusion condition. HC indicates hypercholesterolemia; HeFH, heterozygous familial hypercholesterolemia; HoFH, homozygous familial hypercholesterolemia; and LDL‐C, low‐density lipoprotein cholesterol.

The median observational period of this study was 728 days. Of the 3765 patients enrolled in the study, 3724 were included in the safety analysis group (HoFH, n=108; HeFH, n=2009; hypercholesterolemia with high risk, n=1607; Figure 1); the mean±SD age of the patients was lower in the HoFH cohort (53.9±16.4 years) than in the HeFH (60.7±13.1 years) and hypercholesterolemia with high risk (67.1±11.4 years) cohorts. Most patients were men (HoFH, 59.3%; HeFH, 62.2%; hypercholesterolemia with high risk, 71.6%) and were treated in the outpatient setting (HoFH, 92.6%; HeFH, 91.3%; hypercholesterolemia with high risk, 83.1%). The mean±SD baseline LDL‐C levels were the highest in the HoFH cohort (188.3±108.0 mg/dL) compared with the HeFH (138.9±58.3 mg/dL) and hypercholesterolemia with high risk (99.3±49.3 mg/dL) cohorts.

Major cardiovascular risk factors included a history of CAD, hypertension, and diabetes (Table 1). The history of CAD was higher in the hypercholesterolemia with high risk cohort (86.6%) than in the HoFH (68.5%) and HeFH (75.1%) cohorts. Most patients used concomitant statins, with rosuvastatin being the most common (HoFH, 56.5%; HeFH, 53.5%; hypercholesterolemia with high risk, 43.7%; Table S1). Moderate‐intensity rosuvastatin use was similar among the cohorts (HoFH, 29.6%; HeFH, 34.2%; hypercholesterolemia with high risk, 40.3%), whereas high‐intensity rosuvastatin use was higher in the HoFH (27.6%) and HeFH (21.2%) cohorts than in the hypercholesterolemia with high risk cohort (6.5%). The statin treatment intensities are presented in Table S2.

**Table 1:** Baseline Demographic and Clinical Characteristics of the Safety Analysis Group

Variable	Overall (N=3724)	HoFH (N=108)	HeFH (N=2009)	Hypercholesterolemia with high risk (N=1607)
Male, n (%)	2465 (66.2)	64 (59.3)	1250 (62.2)	1151 (71.6)
Age, y				
Mean±SD	63.2±13.0	53.9±16.4	60.7±13.1	67.1±11.4
Median (quartile 1–quartile 3)	65.0 (54.0–73.0)	55.0 (48.0–64.5)	62.0 (51.0–71.0)	69.0 (60.0–76.0)
Body mass index, kg/m^2^, mean (SD)				
Mean±SD	24.9±4.0	24.1±4.0	24.9±4.1	24.8±3.9
Median (quartile 1–quartile 3)	24.5 (22.1–27.0)	23.6 (21.4–26.8)	24.4 (22.2–27.1)	24.6 (22.2–26.9)
Disease duration, y				
Mean±SD	12.3±7.4	17.1±5.5	14.2±7.0	8.1±6.4
Median (quartile 1–quartile 3)	13.0 (5.3–20.0)	20.0 (17.5–20.0)	20.0 (8.2–20.0)	6.7 (2.6–12.0)
Visit type at the start of treatment, n (%)				
Hospitalization	453 (12.2)	8 (7.4)	174 (8.7)	271 (16.9)
Outpatient clinic	3271 (87.8)	100 (92.6)	1835 (91.3)	1336 (83.1)
LDL‐C (mg/dL) at baseline				
Mean±SD	123.3±61.0	188.3±108.0	138.9±58.3	99.3±49.3
Median (quartile 1–quartile 3)	116.0 (85.0–156.0)	172.0 (124.0–226.0)	133.0 (103.0–170.9)	94.0 (69.0–126.0)
Total cholesterol (mg/dL) at baseline				
Mean±SD	200.9±68.4	259.9±117.5	217.5±64.9	174.4±57.0
Median (quartile 1–quartile 3)	192.0 (157.0–239.0)	241.0 (195.0–291.5)	208.0 (176.0–252.0)	167.0 (138.5–203.0)
Triglyceride (mg/dL) at baseline				
Mean±SD	150.6±99.3	136.7±121.3	147.8±97.8	155.0±99.5
Median (quartile 1–quartile 3)	126.0 (87.0–183.0)	97.5 (73.5–180.0)	123.0 (85.0–178.0)	130.0 (92.0–188.0)
HDL‐C (mg/dL) at the initiation of treatment				
Mean±SD	52.3±15.4	47.1±15.6	52.0±15.1	53.0±15.7
Median (quartile 1–quartile 3)	50.0 (42.0–60.0)	46.0 (35.0–55.5)	50.0 (42.0–60.0)	51.0 (42.0–60.6)
Non–HDL‐C (mg/dL)* at the beginning of treatment				
Mean±SD	148.4±67.5	212.3±118.9	165.7±63.6	120.9±54.8
Median (quartile 1–quartile 3)	141.0 (105.0–184.0)	196.0 (151.0–242.0)	157.0 (125.0–200.0)	114.5 (87.0–150.0)
Comorbidities, n (%)				
Chronic kidney disease	716 (19.2)	9 (8.3)	256 (12.7)	451 (28.1)
Dementia	37 (1.0)	1 (0.9)	18 (0.9)	18 (1.1)
Diabetes	1376 (36.9)	14 (13.0)	599 (29.8)	763 (47.5)
Glucose intolerance	199 (5.3)	7 (6.5)	125 (6.2)	67 (4.2)
Family history of premature CAD	746 (20.0)	29 (26.9)	638 (31.8)	79 (4.9)
History of CAD	2974 (79.9)	74 (68.5)	1508 (75.1)	1392 (86.6)
Hypertension	2626 (70.5)	55 (50.9)	1294 (64.4)	1277 (79.5)
Low HDL‐C (<40 mg/dL)	672 (18.0)	41 (38.0)	338 (19.3)	243 (15.1)
Liver function at treatment initiation				
Normal	2974 (79.9)	71 (65.7)	1587 (79.0)	1316 (81.9)
Mild disorder	682 (18.3)	33 (30.6)	392 (19.5)	257 (16.0)
Moderate impairment	38 (1.0)	2 (1.9)	18 (0.9)	18 (1.1)
Severe disability	10 (0.3)	1 (0.9)	4 (0.2)	5 (0.3)
Unknown	20 (0.5)	1 (0.9)	8 (0.4)	11 (0.7)
Noncardiogenic cerebral infarction	294 (7.9)	2 (1.9)	121 (6.0)	171 (10.6)
Peripheral arterial disease	532 (14.3)	17 (15.7)	200 (10.0)	315 (19.6)
Renal function at treatment initiation				
Normal	2885 (77.5)	92 (85.2)	1680 (83.6)	1113 (69.3)
Mild disorder	606 (16.3)	14 (13.0)	245 (12.2)	347 (21.6)
Moderate impairment	153 (4.1)	1 (0.9)	59 (2.9)	93 (5.8)
Severe disability	64 (1.7)	0 (0.0)	20 (1.0)	44 (2.7)
Unknown	16 (0.4)	1 (0.9)	5 (0.2)	10 (0.6)
Statin intolerance, n (%)	345 (9.3)	2 (1.9)	118 (5.9)	225 (14.0)

CAD indicates coronary artery disease; HDL‐C, high‐density lipoprotein cholesterol; HeFH, heterozygous familial hypercholesterolemia; HoFH, homozygous familial hypercholesterolemia; and LDL‐C, low‐density lipoprotein cholesterol.

* Total cholesterol minus HDL‐C.

Other common concomitant medications included antiplatelets (HoFH, 62.0%; HeFH, 68.5%; hypercholesterolemia with high risk, 77.0%) and antihypertensives (HoFH, 50.0%, HeFH, 64.4%, and hypercholesterolemia with high risk, 71.3%, respectively; Table S1). A total of 2797 patients were eligible for effectiveness analysis group 1 (HoFH, n=91; HeFH, n=1615; hypercholesterolemia with high risk, n=1091; Figure 1). Effectiveness analysis group 2 comprised 2371 patients with on‐label use only (HoFH, n=41; HeFH, n=1342; hypercholesterolemia with high risk, n=988). The baseline characteristics for both effectiveness groups were similar to those for the safety analysis group (Tables S3 and S4).

### Safety Outcomes

#### Serious Adverse Events

Overall, 382 patients within the safety analysis group had at least 1 SAE, with an incidence rate of 10.3% (Table 2). Cardiac disorders were the most common (n=176 [4.7%]), of which the predominant SAEs were angina pectoris (n=84 [2.3%]), myocardial infarction (n=21 [0.6%]), heart failure (n=17 [0.5%]), atrial fibrillation (n=11 [0.3%]), and myocardial ischemia (n=9 [0.2%]). Cerebral infarction and thrombotic cerebral infarction occurred in 9 (0.2%) patients and 1 (<0.1%) patient, respectively. Cerebellar hemorrhage (n=1 [<0.1%]), cerebral hemorrhage (n=5 [0.1%]), subarachnoid hemorrhage (n=4 [0.1%]), transient ischemic attack (n=1 [<0.1%]), and thalamic hemorrhage (n=1, <0.1%) were uncommon SAEs. The incidence rates of myocardial infarction and stroke defined by the Food and Drug Administration custom query were 0.7% (n=25) and 0.3% (n=11), respectively.

**Table 2:** SAEs and Serious ADRs Over 104 Weeks of Follow‐up in the Safety Analysis Group

	SAE (N=3724)			Serious ADR (N=3724)		
	No.	Incidence %*	Incidence	No.	Incidence %*	Incidence
Total number of patients	382	10.3	581	19	0.5	25
Infections and infestations	36	1.0	49	0	0	0
Neoplasms including cysts/polyps†	48	1.3	53	0	0	0
Blood and lymphatic system disorders	4	0.1	4	0	0	0
Immune system disorders	2	0.1	2	0	0	0
Endocrine disorders	3	0.1	3	1	<0.1	1
Metabolism and nutrition disorders	9	0.2	9	0	0	0
Psychiatric disorders	1	<0.1	1	0	0	0
Nervous system disorders	30	0.8	35	2	0.1	2
Cerebellar hemorrhage	1	<0.1	1	0	0	0
Cerebral hemorrhage	5	0.1	s5	0	0	0
Cerebral infarction	9	0.2	9	2	0.1	2
Subarachnoid hemorrhage	4	0.1	4	0	0	0
Transient ischemic attack	1	<0.1	1	0	0	0
Thalamic hemorrhage	1	<0.1	1	0	0	0
Thrombotic cerebral infarction	1	<0.1	1	0	0	0
Other‡	11	0.3	13	0	0	0
Eye disorders	3	0.1	3	0	0	0
Ear and labyrinth disorders	1	<0.1	1	0	0	0
Cardiac disorders (total)	176	4.7	222	3	0.1	3
Acute myocardial infarction	1	<0.1	1	0	0	0
Angina pectoris	84	2.3	95	3	0.1	3
Aortic stenosis	8	0.2	8	0	0	0
Arteriosclerosis coronary artery	2	0.1	2	0	0	0
Atrial fibrillation	11	0.3	12	0	0	0
Heart failure	17	0.5	19	0	0	0
Acute heart failure	4	0.1	4	0	0	0
Chronic heart failure	6	0.2	8	0	0	0
Congestive heart failure	8	0.2	8	0	0	0
CAD	3	0.1	3	0	0	0
Coronary stenosis	2	0.1	2	0	0	0
Myocardial infarction	21	0.6	21	0	0	0
Myocardial ischemia	9	0.2	9	0	0	0
Ventricular fibrillation	4	0.1	5	0	0	0
Ventricular tachycardia	3	0.1	3	0	0	0
Other§	22	0.6	22	0	0	0
Vascular disorders	27	0.7	33	5	0.1	6
Aortic dissection	3	0.1	3	1	<0.1	1
Arteriosclerosis	4	0.1	4	1	<0.1	1
Behcet syndrome	1	<0.1	1	1	<0.1	1
Peripheral ischemia	1	<0.1	1	0	0	0
Vasculitis	1	<0.1	1	1	<0.1	1
Iliac artery stenosis	1	<0.1	1	1	<0.1	1
Peripheral arterial occlusive disease	10	0.3	14	1	<0.1	1
Other**	8	0.2	8	1	<0.1	1
Respiratory, thoracic, and mediastinal disorders	9	0.2	10	1	<0.1	2
Gastrointestinal disorders	27	0.7	32	1	<0.1	1
Hepatobiliary disorders	16	0.4	20	0	0	0
Skin and subcutaneous tissue disorders	1	<0.1	1	0	0	0
Musculoskeletal and connective tissue disorders	7	0.2	8	3	0.1	4
Renal and urinary disorders	18	0.5	18	2	0.1	2
Reproductive system and breast disorders	1	<0.1	1	0	0	0
General disorders and administration site conditions††	27	0.7	31	2	0.1	2
Abnormal laboratory tests	9	0.2	10	2	0.1	2
Injury, poisoning, and procedural complications	22	0.6	34	0	0	0
Surgical and medical procedures	1	<0.1	1	0	0	0

ADR indicates adverse drug reaction; AE, adverse event; CAD, coronary artery disease; and SAE, serious adverse event.

* Incidence (%)=Number of patients with ADR or AE divided by number of subjects included in the safety analysis×100.

^†^ Benign, malignant, or unspecified.

^‡^ Includes the following SAEs: carotid artery stenosis, dementia of Alzheimer type, loss of consciousness, myelopathy, syncope, peripheral neuropathy, epilepsy, and brain edema.

^§^ Includes the following SAEs: arrhythmia, bradycardia, coronary artery aneurysm, Prinzmetal angina, atrial flutter, atrial tachycardia, complete atrioventricular block, cardiac tamponade, cardiopulmonary arrest, cardiogenic shock, low cardiac output syndrome, mitral regurgitation, cardiovascular insufficiency, sinus node dysfunction, and unstable angina.

** Includes the following SAEs: aortic aneurysm, ruptured aortic aneurysm, aortic stenosis, Takayasu arteritis, shock, peripheral artery aneurysm, and peripheral vascular disorders.

^††^ Includes the following SAEs: asthenia, chest discomfort, chest pain, death, edema peripheral, sudden death, inflammation, vascular stent thrombosis, stenosis, and vascular stenosis.

#### Serious ADRs

A total of 19 patients in the safety analysis group had a serious ADR, with an incidence rate of 0.5% (Table 2). Angina pectoris and cerebral infarction occurred in 3 (0.1%) and 2 (0.1%) patients, respectively.

#### AEs and ADRs

Overall, patients more often experienced an AE (HoFH, n=32 [29.6%]; HeFH, n=504 [25.1%]; hypercholesterolemia with high risk, n=405 [25.2%]) than an ADR (HoFH, n=7 [6.5%]; HeFH, n=124 [6.2%]; hypercholesterolemia with high risk, n=74 [4.6%]; Tables S5 and S6). The most common AEs were cardiac disorders (HoFH, n=14 [13.0%]; HeFH, n=127 [6.3%]; hypercholesterolemia with high risk, n=118 [7.3%]), followed by general disorders and administration site conditions (HoFH, n=4 [3.7%]; HeFH, n=69 [3.4%]; hypercholesterolemia with high risk, n=44 [2.7%]). The most common ADRs were general disorders and administration site conditions (HoFH, n=1 [0.9%]; HeFH, n=41 [2.0%]; hypercholesterolemia with high risk, n=19 [1.2%]), followed by laboratory tests (HoFH, n=1 [0.9%]; HeFH, n=21 [1.1%]; hypercholesterolemia with high risk, n=14 [0.9%]) and skin and subcutaneous tissue disorders (HoFH, n=1 [0.9%]; HeFH, n=17 [0.9%]; hypercholesterolemia with high risk, n=15 [0.9%]).

#### SAEs According to the Achieved LDL‐C Levels

A total of 83 (11.7%), 85 (12.4%), and 186 (9.8%) SAEs were experienced by patients with the achieved LDL‐C levels at 12 or 24 weeks of <25 mg/dL, >25 to <40 mg/dL, and ≥40 mg/dL, respectively. There were no trends between the achieved LDL‐C levels and SAEs of interest (injection site reaction, potential drug‐related allergic reaction, muscle‐related events, rhabdomyolysis/myopathy event, diabetes‐related events, cataract‐related AEs, hemorrhagic stroke, neurocognitive events; Table S7).

### Change in LDL‐C

**Figure 2:**
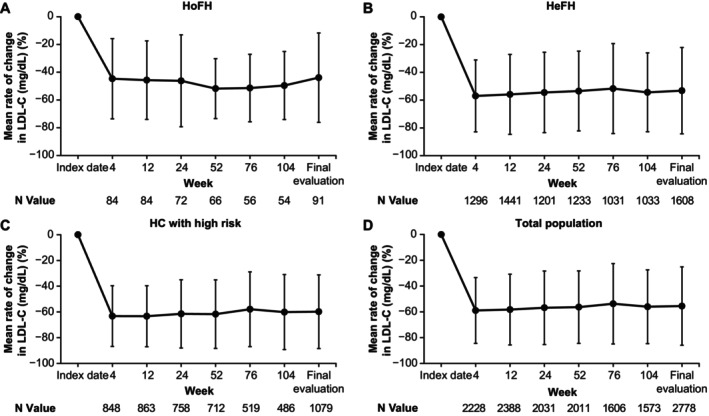
Percentage change (mean±SD) in LDL‐C level from baseline in effectiveness analysis group 1 for patients with (A) HoFH, (B) HeFH, and (C) HC with high risk, and (D) the total population. All cohorts exhibited a significant and sustained reduction in LDL‐C levels over time. *P*<0.001 was calculated for HoFH, HeFH, and hypercholesterolemia with high risk at each follow‐up time point compared with the index date. Statistical analyses were performed using 2‐sided paired *t* tests. N values represent the number of patients who had both LDL‐C values at baseline and any time point after the treatment. HC indicates hypercholesterolemia; HeFH, heterozygous familial hypercholesterolemia; HoFH, homozygous familial hypercholesterolemia; and LDL‐C, low‐density lipoprotein cholesterol.

In effectiveness analysis group 1, at the 12‐week follow‐up, the mean±SD percentage change in LDL‐C from baseline was reduced by 45.7%±28.2 in the HoFH cohort, 55.9%±28.8 in the HeFH cohort, and 63.3%±23.7 in the hypercholesterolemia with high risk cohort (*P*<0.001; Figure 2). Similar trends were observed in effectiveness analysis group 2 (Figure S1). In total, 74.1% of the patients (HoFH, 42.9%; HeFH, 65.5%; hypercholesterolemia with high risk, 91.7%) in effectiveness analysis group 1 achieved the target LDL‐C level at 12 weeks (Table 3). At 104 weeks of follow‐up, the mean±SD percentage change in LDL‐C level from baseline was reduced by 49.6%±24.5 in the HoFH cohort, 54.4%±28.4 in the HeFH cohort, and 60.1%±29.2 in the hypercholesterolemia with high risk cohort (Figure 2). In total, 72.3% of the patients (HoFH, 48.1%; HeFH, 64.4%; hypercholesterolemia with high risk, 91.8%) achieved the target LDL‐C levels at 104 weeks (Table 3). At 104 weeks, the mean±SD percentage change in the total cholesterol, triglycerides, and non–high‐density lipoprotein cholesterol levels were reduced by 36.0%±19.9, 4.0%±73.4, and 50.8%±25.4, respectively. The mean±SD percentage change in high‐density lipoprotein cholesterol levels increased by 12.1%±23.0 in the overall cohort (Table S8). The mean LDL‐C values and percentage change at each time point in effectiveness analysis group 2 are provided in Table S9 and Figure S1. The percentage reductions in LDL‐C in effectiveness analysis group 2 at 104 weeks for HoFH, HeFH, and hypercholesterolemia with high risk were 46.2%, 59.2%, and 63.5%, respectively.

**Table 3:** LDL‐C Control Target Achievement Rate* in Effectiveness Analysis Group 1

Time point	Overall		HoFH		HeFH		Hypercholesterolemia with high risk	
	No.†	Achieved N (%)	No.†	Achieved N (%)	No.*	Achieved N (%)	No.†	Achieved N (%)
Before treatment initiation	2792	535 (19.2)	91	2 (2.2)	1611	41 (2.5)	1090	492 (45.1)
4 wks	2229	1682 (75.5)	84	38 (45.2)	1297	866 (66.8)	848	778 (91.7)
12 wks	2389	1771 (74.1)	84	36 (42.9)	1442	944 (65.5)	863	791 (91.7)
24 wks	2034	1495 (73.5)	72	30 (41.7)	1204	771 (64.0)	758	694 (91.6)
52 wks	2015	1473 (73.1)	66	32 (48.5)	1236	777 (62.9)	713	664 (93.1)
76 wks	1606	1148 (71.5)	56	27 (48.2)	1031	652 (63.2)	519	469 (90.4)
104 wks	1575	1139 (72.3)	54	26 (48.1)	1035	667 (64.4)	486	446 (91.8)
Final evaluation	2783	2034 (73.1)	91	40 (44.0)	1612	1006 (62.4)	1080	988 (91.5)

CAD indicates coronary artery disease; HeFH, heterozygous familial hypercholesterolemia; HoFH, homozygous familial hypercholesterolemia; and LDL‐C, low‐density lipoprotein cholesterol.

* LDL‐C control targets for primary prevention (<100 mg/dL for HoFH, HeFH, and hypercholesterolemia; <120 mg/dL for high risk) and secondary prevention for those with prior CAD/noncardiogenic cerebral infarction (<70 mg/dL for HoFH and HeFH; <100 mg/dL for hypercholesterolemia), those with prior CAD and FH, diabetes, or noncardiogenic cerebral infarction (<70 mg/dL), or those with other secondary prevention (<100 mg/dL) were considered on the basis of the Guideline for Prevention of Arteriosclerotic Diseases, 2022.

^†^ Cases with unknown LDL‐C control target categories were excluded from tabulation.

### Treatment Behavior Change

Between 2016 and 2020, patients initiating evolocumab initially predominantly comprised mostly those with HeFH in 2016 (84.1%) but shifted to those with hypercholesterolemia with high risk in 2020 (60.4%; Table S10). The baseline LDL‐C levels when analyzed according to the year of evolocumab initiation increased in 2017 and mostly decreased gradually year by year (Table S11). A total of 1200 (32.2%) patients discontinued the drug. The most common reasons for discontinuation of evolocumab were patient preference (31.1%), symptom remission or recovery (22.0%), AE (15.5%), and hospital transfer (13.5%; Table S12).

## Discussion

Evidence‐based data on the safety and effectiveness of evolocumab have not been widely reported in patients with HoFH, HeFH, or hypercholesterolemia in a Japanese population. This real‐world surveillance study demonstrated a long‐term, sustained reduction in LDL‐C levels and a good safety profile at 104 weeks after evolocumab initiation (Figure S2). It also provides evidence for the favorable benefit–risk balance of evolocumab in patients with FH and hypercholesterolemia with high risk in the Japanese population.

This real‐world study demonstrated a low rate of SAEs (10.3%) among patients with HoFH, HeFH, and hypercholesterolemia with high risk receiving evolocumab, similar to the previously published results in the subgroup of Japanese patients of the OSLER studies (18.6%).^21^ The low overall incidence rates of myocardial infarction (0.7%) and stroke (0.3%) reported in this study are consistent with those of a previous randomized controlled trial in which PCSK9 inhibition with evolocumab reduced the overall rate of myocardial infarction and stroke in patients with atherosclerotic CVD.^16^ The number of CVD events in Asian populations has recently been published for both women (2.5 events per 1000 person‐years) and men (5.1 events per 1000 person‐years),^22^ which also may explain the low incident rate of cardiovascular events reported in the current study.

Early epidemiological studies have raised concerns regarding the association of low LDL‐C levels with an increased risk of hemorrhagic stroke within a Japanese population.^23^ In this study, no trend was observed between an increased rate of SAEs and the lowest achieved LDL‐C category (<25 mg/dL), suggesting that low LDL‐C levels observed after evolocumab treatment are not associated with SAEs such as hemorrhagic stroke in real‐world practice. In the FOURIER trial, there were no safety concerns with very low LDL‐C levels (<20 mg/dL) over a median of 2.2 years.^24^ Furthermore, there was no excess of SAEs such as hemorrhagic stroke with evolocumab versus placebo arms in Asians compared with other populations.^25^ Similar to our study, FOURIER‐OLE (Further Cardiovascular Outcomes Research With PCSK9 Inhibition in Subjects With Elevated‐Risk–Open Label Extension) demonstrated that the frequency of AEs such as hemorrhagic stroke did not increase over >8 years of follow‐up among patients on evolocumab and did not exceed the rates observed in the placebo arm during the parent study.^26^ The data from our study add to the evidence supporting the safety of evolocumab in Japanese patients who achieve very low LDL‐C levels.

In the present study, a sustained and substantial reduction in LDL‐C levels was observed over 104 weeks of follow‐up for patients with HoFH (−49.6%), HeFH (−54.4%), and hypercholesterolemia with high risk (−60.1%), combined with a low overall rate of adverse outcomes. Importantly, findings of this study indicate that the proportion of patients who achieved the target LDL‐C goal at 104 weeks was notably higher among those with hypercholesterolemia with high risk (91.8%) than among those with HoFH (48.1%) or HeFH (64.4%).

FH is one of the most common monogenic diseases, with variants in 3 genes (*LDL‐R*, *APOB*, and *PCSK9*) identified as its major causes; however, in some probands, the FH phenotype is associated with variants in other genes. Alternatively, polygenic FH with the typical clinical presentation of FH can result from the accumulation of common cholesterol‐increasing alleles.^27^ Therefore, these genetic variations may influence the response to evolocumab among patients with HoFH, HeFH, and hypercholesterolemia with high risk. In our study, the incidence of AEs, particularly cardiac disorders, tended to be higher in patients with HoFH (29.6%) than in those with HeFH (25.1%) and hypercholesterolemia with high risk (25.2%). HoFH is a severe disease, characterized by high LDL‐C levels. In our study, baseline LDL‐C levels were higher in patients with HoFH, and LDL‐C reduction by evolocumab was weaker in these patients than in those with HeFH and hypercholesterolemia with high risk, suggesting that LDL‐C accumulation may be associated with AEs such as cardiac disorders. Importantly, the percentage reduction in LDL‐C in effectiveness analysis group 2 (“on‐label”–only group) was numerically better (HoFH, 46.2%; HeFH, 59.2%; and hypercholesterolemia with high risk, 63.5%) than that in effectiveness analysis group 1. Effectiveness analysis group 1 included patients who initiated evolocumab at low doses and had longer treatment interval, which explains why LDL‐C reduction was weaker in this group than in effectiveness analysis group 2.

In our study, HoFH patients were treated with a moderate‐intensity (65.3%) or high‐intensity statin (33.7%) at baseline, and the baseline LDL‐C level in patients with HoFH was much lower than that reported in previous literature.^28^, ^29^ These results could be related to differences in the effect of evolocumab between the Asian and other populations. The FOURIER Asian population subanalysis showed that evolocumab lowered LDL‐C more significantly in Asians than in other populations (66% versus 58%; *P*<0.001). While the exact cause of this difference is unknown, it may be related to differences in drug metabolism, PCSK9 levels, response to background statin therapy, and concomitant use of other LDL‐C–lowering therapies.^25^

In this study, the proportion of patients who initiated evolocumab increased within the hypercholesterolemia with high risk cohort but decreased within the HeFH cohort over time. Concurrently, baseline LDL‐C levels decreased in the hypercholesterolemia with high risk cohort. These results may reflect the 2017 Japan Atherosclerosis Society guidelines, which recommended an LDL‐C target of <70 mg/dL for patients with high‐risk conditions, potentially influencing physician choice to initiate evolocumab for patients with hypercholesterolemia with high risk, particularly those who did not achieve the recommended LDL‐C goal.^10^ Real‐world data, however, have shown that the proportion of achievement of a target LDL‐C goal for patients with high‐risk conditions, such as FH, acute coronary syndrome, and diabetes, has remained low.^30^ In the present study, the proportion of patients who discontinued evolocumab due to AEs was 15.5%, with discontinuation most often being attributed to patient preference (31.1%), unrelated to AEs. Real‐world data in the United States and European Union have shown high persistence with PCSK9 inhibitors. In the prospective, observational GOULD (Getting to an Improved Understanding of Low‐Density Lipoprotein‐Cholesterol and Dyslipidemia Management) study, 92% of patients continued to receive PCSK9 inhibitors after 2 years.^31^ Similarly, 93% of patients in the prospective, observational cHaractEristics of hYperlipidaeMic pAtieNts at initiation of evolocumab and treatment patternS (HEYMANS) study persisted with evolocumab after 12 months, with the most common reasons for discontinuation being ADRs (3%) and patient request (3%).^32^ Collectively, these results indicate that patient request is a common reason for discontinuation of evolocumab, suggesting that patient education regarding LDL‐C management is needed.

Patients were more often men in all cohorts. Those with HoFH were younger and had a higher level of LDL‐C at baseline than those with HeFH and hypercholesterolemia with high risk. Patients with HoFH or HeFH also had a longer duration of disease, suggesting that patients with FH received earlier diagnoses. Comorbidities, such as chronic kidney disease, diabetes, and hypertension, occurred at a higher frequency at baseline among patients with hypercholesterolemia with high risk than among those with HoFH or HeFH, indicating that when treating patients with hypercholesterolemia with high risk, physicians in Japan may consider comorbidities when choosing more intensive lipid‐lowering regimes. These results were almost consistent with those shown in the interim study of this surveillance, and the incidences of hypertension and diabetes were higher among patients with hypercholesterolemia with high risk than among those with FH.^33^

Of note, there exist differences in indication of evolocumab between Japan and the United States. While the indications in Japan are more disease‐centric, the focus on evolocumab in the United States is to reduce LDL‐C levels as adjunct therapy and outcome reduction.^34^, ^35^ In Japan, evolocumab is indicated for FH and hypercholesterolemia in patients who are both at high risk of cardiovascular events and have shown inadequate response to or are unsuitable (side effects or contraindication) for statins.^34^ These differences may potentially influencing physician behavior to prescribe evolocumab for patients with FH and hypercholesterolemia with high risk.

Overall, there is a lack of evidence regarding evolocumab use in the Asian population. While the efficacy of statins on LDL‐C reduction appears broadly similar between the Asian and White populations, a higher incidence of statin‐induced side effect has been observed in the Asian population.^36^ This study showed consistent LDL‐C reduction and improvements in the achievements rates of target LDL‐C levels with evolocumab in the Asian population, with no safety concerns. Therefore, this study helps to provide a good solution to achieve target LDL‐C levels in Asian patients who cannot continue statin treatment due to safety problems.

### Study Limitations

First, the study design did not include a placebo arm, and missing/incomplete data and loss to follow‐up were inevitable in the real‐world practice. Second, given this data type with a lack of homogeneity, prescription bias, selection bias, information bias, and compounding factors are possible. Third, the diagnoses of FH were made by physicians in accordance with the Japan Atherosclerosis Society FH diagnostic criteria. Additionally, HeFH diagnosis was not confirmed through genetic testing in most patients. Fourth, the generalizability of the findings is limited to the Japanese population, as this study included only Japanese patients. Finally, because this study evaluated real‐world data, some patients had achieved the target LDL‐C level before the administration of evolocumab. However, because evolocumab was administered to patients who were at high risk of cardiovascular events and required further LDL‐C reduction, it was meaningful to continue analyzing the target achievement rate in these patients. Furthermore, it was difficult to verify the effect of evolocumab alone in a real‐world observational study, which is considered to be a limitation.

### Conclusions

Overall, this study indicated the favorable benefit–risk balance of evolocumab in patients with FH and hypercholesterolemia with high risk. In the given patient population and taking into account the study design limitations, evolocumab is well tolerated and effectively reduces LDL‐C levels in patients with HoFH, HeFH, and hypercholesterolemia with high risk in Japan.

## Sources of Funding

This study was funded by Amgen K.K., Tokyo, Japan, and Astellas Pharma Inc., Tokyo, Japan.

## Disclosures

Dr Yokote reports speaker fees from Astellas, AstraZeneca, MSA, Ono, Kowa, Sanofi, Daiichi Sankyo, Taisho, Sumitomo Dainippon, Takeda, Mitsubishi Tanabe, Eli Lilly Japan, Boehringer Ingelheim Japan, Novartis, Novo Nordisk, and Janssen; research funding from Taisho; and scholarship funding from Astellas, MSD, Ono, Kowa, Shionogi, Daiichi Sankyo, Taisho, Sumitomo Dainippon, Takeda, Mitsubishi Tanabe, Teijin, Nippon Boehringer Ingelheim, Novo Nordisk, and Bayer. Dr Ako reports speaker fees from Amgen, Kowa, Bayer, Pfizer, AstraZeneca, and Daiichi Sankyo. Dr Kitagawa reports payment or honoraria from Daiichi Sankyo, Amgen, and Kowa. N.O. is an employee of Amgen. Dr Sheng is an employee and a stockholder of Amgen. M.S. is an employee of Amgen. Dr Teramoto has no disclosures to report.

## Supporting information

Data S1
