# Supplementary material for: Safety and Effectiveness of Low‐Density Lipoprotein Cholesterol–Lowering Therapy With Evolocumab for Familial Hypercholesterolemia/Hypercholesterolemia in Japan: A Real‐World, Postmarketing, Single‐Arm Study
Source: J Am Heart Assoc. 2024 Oct 29;13(21):e035809. doi: 10.1161/JAHA.124.035809 (PMC11935657; doi:10.1161/JAHA.124.035809)
Supplement: Supplementary file 1 — Data S1 [file JAH3-13-e035809-s001.pdf]

SUPPLEMENTAL MATERIAL

Table S1

Concomitant drug use in the safety analysis group.

Medication, n (%)	Overall (N=3724)	HoFH (N=108)	HeFH (N=2009)	HC with high risk (N=1607)
**None**	49 (1.3)	2 (1.9)	18 (0.9)	29 (1.8)
**Total patients with concomitant drugs**	3674 (98.7)	106 (98.1)	1991 (99.1)	1577 (98.1)
**Angina treatment**	2119 (56.9)	49 (45.4)	1092 (54.4)	978 (60.9)
**Anticoagulants**	356 (9.6)	12 (11.1)	172 (8.6)	172 (10.7)
**Antiplatelet drugs**	2681 (72.0)	67 (62.0)	1376 (68.5)	1238 (77.0)
**Antihypertensive drugs**	2494 (67.0)	54 (50.0)	1294 (64.4)	1146 (71.3)
**Diabetes medication**	888 (23.8)	10 (9.3)	382 (19.0)	496 (30.9)
**Other**	2425 (65.1)	52 (48.1)	1208 (60.1)	1165 (72.5)
**HC therapeutic agent (statins)**				
Total	3388 (91.0)	103 (95.4)	1894 (94.3)	1391 (86.6)
Atorvastatin	926 (24.9)	27 (25.0)	531 (26.4)	368 (22.9)
Fluvastatin	38 (1.0)	0 (0.0)	18 (0.9)	20 (1.2)
Pitavastatin	638 (17.1)	17 (15.7)	324 (16.1)	297 (18.5)
Pravastatin	164 (4.4)	4 (3.7)	73 (3.6)	87 (5.4)
Rosuvastatin	1839 (49.4)	61 (56.5)	1075 (53.5)	703 (43.7)
Simvastatin	16 (0.4)	0 (0.0)	7 (0.3)	9 (0.6)
**HC therapeutic agent (nonstatins)**				
Total	2253 (60.5)	73 (67.6)	1349 (67.1)	831 (51.7)
Bezafibrate	46 (1.2)	1 (0.9)	28 (1.4)	17 (1.1)
Colestimide	152 (4.1)	12 (11.1)	123 (6.1)	17 (1.1)
Ezetimibe	1970 (52.9)	64 (59.3)	1216 (60.5)	690 (42.9)
Fenofibrate	40 (1.1)	1 (0.9)	24 (1.2)	15 (0.9)
Lomitapide	11 (0.3)	8 (7.4)	3 (0.1)	0 (0.0)
Pemafibrate	101 (2.7)	1 (0.9)	42 (2.1)	58 (3.6)
Probucol	110 (3.0)	10 (9.3)	90 (4.5)	10 (0.6)
Other	784 (21.1)	29 (26.9)	444 (22.1)	311 (19.4)

HC, hypercholesterolemia; HeFH, heterozygous familial hypercholesterolemia; HoFH, homozygous familial hypercholesterolemia.

Table S2
Pretreatment statin intensity in the safety analysis group.

Medication, n (%)	Overall (N=3028)	HoFH (N=98)	HeFH (N=1734)	HC with high risk (N=1196)
**Low intensity**	115 (3.8)	3 (3.1)	52 (3.0)	60 (5.0)
Simvastatin (10 mg to <20 mg per day)	5 (0.2)	0 (0.0)	3 (0.2)	2 (0.2)
Pravastatin (10 mg to <40 mg per day)	81 (2.7)	3 (3.1)	37 (2.1)	41 (3.4)
Fluvastatin (20 mg to <80 mg per day)	29 (1.0)	0 (0.0)	12 (0.7)	17 (1.4)
**Moderate intensity**	2300 (76.0)	64 (65.3)	1183 (68.2)	1053 (88.0)
Atorvastatin (10 mg to <40 mg per day)	624 (20.6)	20 (20.4)	313 (18.1)	291 (24.3)
Rosuvastatin (5 mg to <20 mg per day)	1104 (36.5)	29 (29.6)	593 (34.2)	482 (40.3)
Pitavastatin (1 mg to <4 mg or less per day)	577 (19.1)	15 (15.3)	281 (16.2)	281 (23.5)
**High intensity**	623 (20.6)	33 (33.7)	504 (29.1)	86 (7.2)
Atorvastatin (≥40 mg to 80 mg or less per day)	151 (5.0)	6 (6.1)	137 (7.9)	8 (0.7)
Rosuvastatin (≥20 mg to <40 mg or less per day)	472 (15.6)	27 (27.6)	367 (21.2)	78 (6.5)
Unknown dosage and administration	4 (0.1)	0 (0.0)	1 (0.0)	3 (0.2)

HC, hypercholesterolemia; HeFH, heterozygous familial hypercholesterolemia; HoFH, homozygous familial hypercholesterolemia.

Table S3

Baseline demographic and clinical characteristics of effectiveness analysis group 1.

Variable	HoFH (N=91)	HeFH (N=1615)	HC with high risk (N=1091)
**Male, n (%)**	52 (57.1)	1021 (63.2)	797 (73.1)
**Age, years**			
Mean±SD	53.6±16.7	60.3±13.1	66.4±11.5
Median (Q1; Q3)	55.0 (47.0; 65.0)	61.0 (51.0; 70.0)	68.0 (59.0; 75.0)
**BMI (kg/m^2), mean (SD)**			
Mean±SD	24.1±4.1	24.9±4.2	25.0±3.8
Median (Q1; Q3)	23.7 (21.3; 26.9)	24.4 (22.3; 27.1)	24.7 (22.4; 27.0)
**Disease duration**			
Mean±SD	17.0±5.5	14.5±6.9	7.6±6.1
Median (Q1; Q3)	20.0 (15.0; 20.0)	20.0 (9.1; 20.0)	6.5 (2.3; 11.0)
**Visit type at treatment initiation, n (%)**			
Hospitalization	8 (8.8)	157 (9.7)	232 (21.3)
Outpatient clinic	83 (91.2)	1458 (90.3)	859 (78.7)
**LDL-C (mg/dL) at baseline**			
Mean±SD	186.7±77.8	146.6±53.4	111.7±43.7
Median (Q1; Q2)	175.2 (135.0; 231.0)	137.4 (111.0; 172.0)	102.0 (83.0; 134.0)
**Total cholesterol (mg/dL) at baseline**			
Mean±SD	257.1±85.7	224.5±60.2	186.9±50.4
Median (Q1; Q2)	244.0 (198.0; 287.0)	213.0 (184.0; 255.0)	178.0 (152.0; 211.0)
**Triglyceride (mg/dL) at baseline**			
Mean±SD	138.6±123.1	149.4±97.2	161.0±100.9
Median (Q1; Q2)	100.0 (75.0; 181.0)	127.0 (86.0; 183.0)	139.0 (99.0; 192.0)
**HDL-C (mg/dL) at treatment initiation**			
Mean±SD	46.7±15.7	51.1±14.8	52.0±15.9
Median (Q1; Q3)	46.0 (35.0; 55.0)	49.0 (41.0; 59.0)	49.2 (41.1; 60.0)
**Non–HDL-C (mg/dL)* at treatment initiation**			
Mean±SD	209.7±85.6	173.4±58.5	134.3±47.7
Median (Q1; Q3)	196.7 (154.5; 238.0)	162.6 (134.0; 202.4)	124.0 (101.0; 159.0)
**Comorbidities**			
Chronic kidney disease	9 (9.9)	192 (11.9)	315 (28.9)
Dementia	1 (1.1)	15 (0.9)	12 (1.1)
Diabetes mellitus	11 (12.1)	472 (29.2)	525 (48.1)
Glucose intolerance	7 (7.7)	99 (6.1)	45 (4.1)
Family history of premature CAD	26 (28.6)	519 (32.1)	61 (5.6)
History of CAD	63 (69.2)	1237 (76.6)	941 (86.3)
Hypertension	49 (53.8)	1038 (64.3)	867 (79.5)
Low HDL-C (<40 mg/dL)	35 (38.5)	335 (20.7)	185 (17.0)
Liver function at treatment initiation			
Normal	61 (67.0)	1262 (78.1)	885 (81.1)
Mild disorder	27 (29.7)	332 (20.6)	183 (16.8)
Moderate impairment	2 (2.2)	16 (1.0)	15 (1.4)
Severe disability	1 (1.1)	2 (0.1)	2 (0.2)

Unknown	0 (0.0)	3 (0.2)	6 (0.5)
Noncardiogenic cerebral infarction	2 (2.2)	98 (6.1)	121 (11.1)
Peripheral arterial disease	13 (14.3)	163 (10.1)	225 (20.6)
Renal function at treatment initiation			
Normal	76 (83.5)	1367 (84.6)	743 (68.1)
Mild disorder	14 (15.4)	189 (11.7)	254 (23.3)
Moderate impairment	1 (1.1)	45 (2.8)	68 (6.2)
Severe disability	0 (0.0)	12 (0.7)	21 (1.9)
Unknown	0 (0.0)	2 (0.1)	5 (0.5)

BMI, body mass index; CAD, coronary artery disease; HC, hypercholesterolemia; HDL-C, high-density lipoprotein cholesterol; HeFH, heterozygous familial hypercholesterolemia; HoFH, homozygous familial hypercholesterolemia; LDL-C, low-density lipoprotein cholesterol; Q, quartile; SD, standard deviation.

* Total cholesterol minus HDL-C.

Table S4

Baseline demographic and clinical characteristics of effectiveness analysis group 2.

Variable	HoFH (N=41)	HeFH (N=1342)	HC with high risk (N=988)
**Male, n (%)**	23 (56.1)	853 (63.6)	724 (73.3)
**Age, years**			
Mean±SD	59.0±13.2	60.5±12.9	66.6±11.4
Median (Q1; Q3)	60.0 (53.0; 67.0)	61.0 (51.0; 70.0)	68.0 (59.0; 75.0)
**Body mass index (kg/m^2), mean (SD)**			
Mean±SD	24.2±4.2	25.0±4.2	24.9±3.8
Median (Q1; Q3)	23.8 (20.9; 26.8)	24.5 (22.2; 27.1)	24.7 (22.3; 27.0)
**Disease duration, years**			
Mean±SD	18.2±3.6	13.8±7.0	7.5±6.1
Median (Q1; Q3)	20.0 (20.0; 20.0)	17.0 (8.0; 20.0)	6.4 (2.2; 10.4)
**Visit type at treatment initiation, n (%)**			
Hospitalization	1 (2.4)	140 (10.4)	192 (19.4)
Outpatient clinic	40 (97.6)	1202 (89.6)	796 (80.6)
**LDL-C (mg/dL) at baseline**			
Mean±SD	171.0±59.7	146.1±53.4	111.2±43.6
Median (Q1; Q2)	164.1 (125.0; 197.0)	137.0 (110.0; 171.4)	102.0 (83.0; 133.0)
**Total cholesterol (mg/dL) at baseline**			
Mean±SD	234.6±52.1	223.6±60.2	186.6±49.6
Median (Q1; Q2)	230.0 (203.0; 257.0)	213.0 (184.0; 254.0)	178.0 (152.0; 211.0)
**Triglyceride (mg/dL) at baseline**			
Mean±SD	137.4±82.9	151.0±97.6	159.2±95.0
Median (Q1; Q2)	113.0 (79.0; 191.0)	130.0 (87.0; 185.0)	139.4 (98.0; 191.0)
**HDL-C (mg/dL) at treatment initiation**			
Mean±SD	44.5±15.7	51.0±15.0	52.2±15.0
Median (Q1; Q3)	44.0 (32.5; 54.0)	49.0 (41.0; 59.0)	49.4 (42.0; 60.0)
**Non–HDL-C (mg/dL)* at treatment initiation**			
Mean±SD	188.4±52.4	172.9±58.5	134.0±47.1
Median (Q1; Q3)	190.5 (162.0; 211.0)	162.5 (133.0; 202.0)	124.0 (101.0; 158.8)
**Comorbidities**			
Chronic kidney disease	4 (9.8)	162 (12.1)	294 (29.8)
Dementia	1 (2.4)	11 (0.8)	11 (1.1)
Diabetes mellitus	4 (9.8)	395 (29.4)	478 (48.4)
Glucose intolerance	2 (4.9)	86 (6.4)	41 (4.1)
Family history of premature CAD	14 (34.1)	441 (32.9)	54 (5.5)
History of CAD	30 (73.2)	1042 (77.6)	875 (88.6)
Hypertension	23 (56.1)	869 (64.8)	800 (81.0)
Low HDL-C (<40 mg/dL)	17 (41.5)	287 (21.4)	162 (16.4)
Liver function at treatment initiation			
Normal	27 (65.9)	1052 (78.4)	813 (82.3)
Mild disorder	14 (34.1)	273 (20.3)	156 (15.8)
Moderate impairment	0 (0.0)	13 (1.0)	13 (1.3)
Severe disability	0 (0.0)	1 (0.1)	1 (0.1)

Unknown	0 (0.0)	3 (0.2)	5 (0.5)
Noncardiogenic cerebral infarction	2 (4.9)	83 (6.2)	114 (11.5)
Peripheral arterial disease	6 (14.6)	137 (10.2)	215 (21.8)
Renal function at treatment initiation			
Normal	36 (87.8)	1137 (84.7)	665 (67.3)
Mild disorder	4 (9.8)	158 (11.8)	239 (24.2)
Moderate impairment	1 (2.4)	37 (2.8)	61 (6.2)
Severe disability	0 (0.0)	8 (0.6)	19 (1.9)
Unknown	0 (0.0)	2 (0.1)	4 (0.4)

BMI, body mass index; CAD, coronary artery disease; HC, hypercholesterolemia; HDL-C, high-density lipoprotein cholesterol; HeFH, heterozygous familial hypercholesterolemia; HoFH, homozygous familial hypercholesterolemia; LDL-C, low-density lipoprotein cholesterol; Q, quartile; SD, standard deviation.
* Total cholesterol minus HDL-C.

Table S5
Adverse events by type in the overall safety analysis group.

	HoFH			HeFH			HC with high risk		
	N	Incidence % (95% CI)	Incidence/1000 person-years	N	Incidence % (95% CI)	Incidence/1000 person-years	N	Incidence % (95% CI)	Incidence/ 1000 person-years
**Total**	32	29.6 (21.2–39.2)	291.1	504	25.1 (23.2–27.0)	240.2	405	25.2 (23.1–27.4)	272.2
**Infections and infestations**	2	1.9 (0.2–6.5)	10.4	45	2.2 (1.6–3.0)	14.9	47	2.9 (2.2–3.9)	23.5
**Neoplasms benign, malignant, and unspecified (including cysts and polyps)**	0	0	0	29	1.4 (1.0–2.1)	8.8	26	1.6 (1.1–2.4)	10.3
**Blood and lymphatic system disorders**	0	0	0	11	0.6 (0.3–1.0)	3.3	14	0.9 (0.5–1.5)	5.5
**Immune system disorders**	0	0	0	3	0.2 (<0.1–0.4)	0.8	0	0	0
**Endocrine disorders**	0	0	0	4	0.2 (0.1–0.5)	1.1	4	0.3 (0.1–0.6)	1.5
**Metabolism and nutrition disorders**	3	2.8 (0.6–7.9)	20.8	40	2.0 (1.4–2.7)	12.4	38	2.4 (1.7–3.2)	15.8
**Psychiatric disorders**	1	0.9 (<0.1–5.1)	5.2	12	0.6 (0.3–1.0)	3.3	4	0.3 (0.1–0.6)	1.5
**Nervous system disorders**	1	0.9 (<0.1–5.1)	5.2	49	2.4 (1.8–3.2)	14.9	28	1.7 (1.2–2.5)	12.5
**Eye disorders**	0	0	0	6	0.3 (0.1–0.7)	1.7	5	0.3 (0.1–0.7)	1.8
**Ear and labyrinth disorders**	0	0	0	4	0.2 (0.1–0.5)	1.1	5	0.3 (0.1–0.72)	1.8
**Cardiac disorders**	14	13.0 (7.3–20.8)	88.4	127	6.3 (5.3–7.5)	42.7	118	7.3 (6.1–8.7)	58.0
**Vascular disorders**	0	0	0	31	1.5 (1.1–2.2)	9.7	25	1.6 (1.0–2.3)	10.3
**Respiratory, thoracic, and mediastinal disorders**	1	0.9 (<0.1–5.1)	5.2	21	1.1 (0.7–1.6)	6.9	14	0.9 (0.5–1.5)	5.5
**Gastrointestinal disorders**	3	2.8 (0.6–7.9)	20.8	44	2.2 (1.6–2.9)	14.3	42	2.6 (1.9–3.5)	20.2
**Hepatobiliary disorders**	6	5.6 (2.1–11.7)	31.2	41	2.0 (1.5–2.8)	11.9	24	1.5 (1.0–2.2)	11.4
**Skin and subcutaneous tissue disorders**	3	2.8 (0.6–7.9)	20.8	31	1.5 (1.1–2.2)	11.0	27	1.7 (1.1–2.4)	10.6
**Musculoskeletal/connective tissue disorders**	2	1.9 (0.2–6.5)	10.4	53	2.6 (2.0–3.3)	17.1	29	1.8 (1.2–2.3)	11.7
**Renal and urinary disorders**	1	0.9 (<0.1–5.1)	5.2	18	0.9 (0.5–1.4)	5.0	21	1.3 (0.8–2.0)	7.7
**Reproductive system and breast disorders**	0	0	0	2	0.1 (<0.1–0.4)	0.6	5	0.3 (0.1–0.7)	1.8

**General disorders/administration site conditions**	4	3.7 (1.0–9.2)	26.0	69	3.43 (2.68–4.33)	27.3	44	2.7 (2.0–3.7)	22.7
**Laboratory tests**	4	3.7 (1.0–9.2)	36.4	66	3.29 (2.55–4.16)	22.1	46	2.9 (2.1–3.8)	23.8
**Injury, poisoning, and procedural complications**	0	0	0	22	1.10 (0.69–1.65)	8.3	26	1.6 (1.1–2.4)	14.3
**Surgical and medical procedures**	1	0.9 (<0.1–5.1)	5.2	4	0.20 (0.05–0.51)	1.1	0	0	0

CI, confidence interval; HC, hypercholesterolemia; HeFH, heterozygous familial hypercholesterolemia; HoFH, homozygous familial hypercholesterolemia.

Table S6
Adverse drug reactions by type in the overall safety analysis group.

	HoFH			HeFH			HC with high risk		
	**N**	**Incidence % (95% CI)**	**Incidence/1000 person-years**	**N**	**Incidence % (95% CI)**	**Incidence/1000 person-years**	**N**	**Incidence % (95% CI)**	**Incidence/1000 person-years**
**Total**	7	6.48 (2.65–12.90)	46.8	124	6.17 (5.16–7.31)	53.5	74	4.60 (3.63–5.75)	36.7
**Infections and infestations**	0	0	0	1	0.05 (0.00–0.28)	0.3	1	0.06 (0.00–0.35)	0.4
**Blood and lymphatic system disorders**	0	0	0	1	0.05 (0.00–0.28)	0.3	0	0	0
**Immune system disorders**	0	0	0	1	0.05 (0.00–0.28)	0.3	0	0	0
**Endocrine disorders**	0	0	0	2	0.10 (0.01–0.36)	0.6	0	0	0
**Metabolism and nutrition disorders**	0	0	0	3	0.15 (0.03–0.44)	0.8	3	0.19 (0.04–0.54)	1.1
**Psychiatric disorders**	0	0	0	4	0.20 (0.05–0.51)	1.1	1	0.06 (0.00–0.35)	0.4
**Nervous system disorders**	0	0	0	11	0.55 (0.27–0.98)	3.0	3	0.19 (0.04–0.54)	1.5
**Eye disorders**	0	0	0	1	0.05 (0.00–0.28)	0.3	0	0	0
**Cardiac disorders**	0	0	0	3	0.15 (0.03–0.44)	0.8	5	0.31 (0.10–0.72)	1.8
**Vascular disorders**	0	0	0	6	0.30 (0.11–0.65)	1.9	1	0.06 (0.00–0.35)	0.4
**Respiratory, thoracic, and mediastinal disorders**	0	0	0	2	0.10 (0.01–0.36)	0.8	0	0	0
**Gastrointestinal disorders**	1	0.93 (0.02–5.05)	5.2	7	0.35 (0.14–0.72)	3.0	5	0.31 (0.10–0.72)	2.9
**Hepatobiliary disorders**	2	1.85 (0.23–6.53)	10.4	10	0.50 (0.24–0.9)	3.3	1	0.06 (0.00–0.35)	0.4
**Skin and subcutaneous tissue disorders**	1	0.93 (0.02–5.05)	10.4	17	0.85 (0.49–1.35)	6.1	15	0.93 (0.52–1.53)	6.2
**Musculoskeletal/connective tissue disorders**	1	0.93 (0.02–5.05)	5.2	15	0.75 (0.42–1.23)	5.5	9	0.56 (0.26–1.06)	3.7
**Renal and urinary disorders**	0	0	0	2	0.10 (0.01–0.36)	0.6	3	0.19 (0.04–0.54)	1.1
**Reproductive system and breast disorders**	0	0	0	1	0.05 (0.00–0.28)	0.3	0	0	0
**General disorders/administration site conditions**	1	0.93 (0.02–5.05)	10.4	41	2.04 (1.47–2.76)	17.7	19	1.18 (0.71–1.84)	9.9
**Laboratory tests**	1	0.93 (0.02–5.05)	5.2	21	1.05 (0.65–1.59)	6.9	14	0.87 (0.48–1.46)	6.2
**Injury, poisoning, and procedural complications**	0	0	0	0	0	0	2	0.12 (0.02–0.45)	0.7

CI, confidence interval; HC, hypercholesterolemia; HeFH, heterozygous familial hypercholesterolemia; HoFH, homozygous familial hypercholesterolemia.

Table S7

SAEs according to the achieved LDL-C levels.

	All patients	Achieved LDL-C category		
	N=3724	<25 mg/dL (N=707)*	≥25 to <40 mg/dL (N=685)*	≥40 mg/dL (N=1894)*
**SAE**	382 (10.3)	83 (11.7)	85 (12.4)	186 (9.8)
**Injection site reaction**	0	0	0	0
**Potential drug-related allergic reaction events**	5 (0.13)	0	0	5 (0.26)
**Muscle-related events**	16 (0.43)	3 (0.42)	1 (0.15)	9 (0.48)
**Rhabdomyolysis/myopathy event**	2 (0.05)	0	0	2 (0.11)
**Diabetes-related events**	9 (0.24)	1 (0.14)	0	8 (0.42)
**Cataract-related AEs**	3 (0.08)	2 (0.28)	0	1 (0.05)
**Cerebral hemorrhage–related events**	14 (0.38)	3 (0.42)	3 (0.44)	6 (0.32)
**Neurocognitive events**	1 (0.03)	0	0	1 (0.05)

AE, adverse event; LDL-C, low-density lipoprotein cholesterol; SAE, serious adverse event.

Data presented as n (%).

* Patients in the safety analysis with post-administration LDL-C data.

Table S8.

Change in lipid levels over time in the effectiveness analysis group 1.

	HoFH		HeFH		HC with high risk		Total population	
	**N**	**Mean±SD**	**N**	**Mean±SD**	**N**	**Mean±SD**	**N**	**Mean±SD**
**TC (mg/dL)**								
4 weeks	76	−30.9±21.8	1021	−37.9±18.2	636	−38.1±17.2	1733	−37.7±18.1
12 weeks	73	−31.8±21.1	1127	−37.2±19.5	653	−37.8±16.6	1853	−37.2±18.6
24 weeks	64	−32.7±25.4	917	−35.5±20.7	581	−37.0±17.6	1562	−35.9±19.8
52 weeks	56	−34.5±18.2	927	−34.7±20.3	537	−36.9±17.7	1520	−35.4±19.4
76 weeks	45	−34.2±20.5	763	−33.8±21.4	377	−36.4±18.0	1185	−34.7±20.4
104 weeks	45	−34.4±18.0	772	−35.2±20.8	360	−38.0±17.9	1177	−36.0±19.9
Final evaluation	81	−30.6±24.9	1273	−34.5±21.4	828	−36.8±19.0	2182	−35.2±20.7
**HDL-C (mg/dl)**								
4 weeks	80	7.3±16.5	1262	7.9±17.1	830	7.6±16.8	2172	7.7±16.9
12 weeks	80	10.1±21.8	1404	10.8±32.4	835	9.5±19.0	2319	10.3±28.0
24 weeks	71	8.4±22.7	1169	12.5±40.7	739	9.2±18.6	1979	11.1±33.6
52 weeks	64	16.3±25.7	1202	12.4±24.8	698	8.8±20.9	1964	11.2±23.6
76 weeks	55	15.5±29.7	999	13.3±23.8	502	8.6±20.6	1556	11.9±23.1
104 weeks	53	20.9±36.9	1005	12.7±22.6	468	9.9±21.3	1526	12.1±23.0
Final evaluation	89	16.0±31.8	1567	12.4±22.4	1057	8.5±22.1	2713	11.0±22.8
**TG (mg/dL)**								
4 weeks	81	0.3±45.8	1252	−8.8± 44.0	823	−9.4±45.3	2156	−8.7±44.6
12 weeks	81	−5.6±39.4	1389	−8.0± 46.9	830	−8.2±57.6	2300	−8.0±50.8
24 weeks	71	−7.9±39.5	1164	−6.3±46.0	732	−7.4±49.4	1967	−6.8±47.1
52 weeks	64	−13.0±34.4	1193	−2.5±49.8	691	−9.3±48.0	1948	−5.3±48.9
76 weeks	55	−13.6±36.2	994	−1.4±54.9	498	−8.6±64.6	1547	−4.2±57.8
104 weeks	53	−12.3±39.0	993	−1.1±83.4	470	−9.1±49.6	1516	−4.0±73.4
Final evaluation	89	−7.2±44.0	1554	−2.2±75.2	1049	−7.2±54.7	2692	−4.3±67.1
**Non–HDL-C (mg/dL)**								
4 weeks	74	−39.6±27.4	999	−51.7±22.3	622	−55.5±22.7	1695	−52.6±22.9
12 weeks	70	−41.5±26.9	1103	−51.3±25.3	637	−56.1±21.6	1810	−52.6±24.3

	HoFH		HeFH		HC with high risk		Total population	
	**N**	**Mean±SD**	**N**	**Mean±SD**	**N**	**Mean±SD**	**N**	**Mean±SD**
24 weeks	63	−43.0±28.0	897	−49.0±26.5	567	−55.0±25.3	1527	−51.0±26.3
52 weeks	54	−46.5±21.3	906	−48.0±26.0	525	−55.0±24.3	1485	−50.4±25.4
76 weeks	44	−45.3±24.0	743	−47.5±28.3	367	−52.1±26.1	1154	−48.9±27.5
104 weeks	44	−46.5±21.5	751	−49.0±25.9	347	−55.1±24.3	1142	−50.8±25.4
Final evaluation	80	−40.7±31.4	1246	−47.8±27.4	815	−53.9±25.3	2141	−49.9±27.0

HC, hypercholesterolemia; HDL-C, high-density lipoprotein cholesterol; HeFH, heterozygous familial hypercholesterolemia; HoFH, homozygous familial hypercholesterolemia; SD, standard deviation; TC, total cholesterol; TG, triglycerides.

Table S9

LDL-C values and percentage change over time in effectiveness analysis group 2.

	Overall			HoFH			HeFH			HC with high risk		
	N	Mean±SD mg/dL	Mean rate of change±SD %	N	Mean±SD mg/dL	Mean rate of change±SD %	N	Mean±SD mg/dL	Mean rate of change±SD %	N	Mean±SD mg/dL	Mean rate of change±SD %
**Prior to initiation**	2118	130.6±52.7		39	170.5±61.2		1252	145.3±53.4		827	106.4±40.5	
**4 weeks**	1689	49.3±37.6	−62.5±23.7	34	98.4±78.5	−46.6±32.2	1009	56.0±38.1	−60.6±24.4	646	36.1±27.3	−66.1±21.3
**12 weeks**	1767	47.3±35.0	−63.0±24.8	34	90.5±64.3	−47.2±30.2	1078	54.0±35.9	−61.0±26.0	655	33.9±24.9	−67.0±21.6
**24 weeks**	1448	48.5±37.1	−61.6±26.4	23	98.1±104.7	−45.6±45.6	861	55.7±35.8	−59.6±26.9	564	35.5±28.3	−65.2±24.0
**52 weeks**	1375	50.1±36.4	−60.3±26.4	23	84.8±60.8	−55.1±19.7	866	57.6±38.1	−57.7±27.6	486	35.1±25.0	−65.3±23.7
**76 weeks**	1089	52.9±36.1	−57.8±30.3	20	86.0±54.5	−52.5±19.7	703	59.2±37.3	−55.8±32.3	366	38.9±27.0	−61.8±26.1
**104 weeks**	1044	50.7±36.1	−60.4±26.4	17	81.0±44.0	−46.2±20.5	699	56.0±35.7	−59.2±25.2	328	38.0±33.0	−63.5±28.9
**Final evaluation**	2118	49.5±38.4	−60.8±28.4	39	98.1±70.8	−42.0±37.9	1252	56.8±38.7	−58.8±29.1	827	36.1±30.1	−64.6±26.2

HC, hypercholesterolemia; HeFH, heterozygous familial hypercholesterolemia; HoFH, homozygous familial hypercholesterolemia; LDL-C, low-density lipoprotein cholesterol; SD, standard deviation.

Table S10

Initiation of evolocumab by diagnosis type and year in the safety analysis group.

Year	Overall<br>N	HoFH<br>n (%)	HeFH<br>n (%)	HC with high risk<br>n (%)
**Overall**	3724	108 (2.9)	2009 (53.9)	1607 (43.2)
**2016**	429	43 (10.0)	361 (84.1)	25 (5.8)
**2017**	703	23 (3.3)	493 (70.1)	187 (26.6)
**2018**	743	14 (1.9)	392 (52.8)	337 (45.4)
**2019**	637	10 (1.6)	301 (47.3)	326 (51.2)
**2020**	1212	18 (1.5)	462 (38.1)	732 (60.4)

HC, hypercholesterolemia; HeFH, heterozygous familial hypercholesterolemia; HoFH, homozygous familial hypercholesterolemia.

Table S11

Changes in the baseline LDL-C levels by evolocumab initiation year in the safety analysis group.

Year	HoFH		HeFH		HC with high risk	
	N	Median (Q1; Q3)	N	Median (Q1; Q3)	N	Median (Q1; Q3)
**Overall**	102	173.5 (125.0; 226.0)	1938	133.0 (103.0; 170.0)	1538	94.0 (69.0; 127.0)
**2016**	41	184.0 (150.0; 236.0)	348	149.0 (124.5; 180.5)	25	111.0 (93.0; 134.8)
**2017**	22	192.0 (175.2; 221.0)	475	141.0 (116.0; 172.0)	185	109.6 (86.0; 133.0)
**2018**	13	158.0 (120.2; 206.0)	380	131.0 (104.5; 171.0)	323	97.0 (75.0; 134.0)
**2019**	9	135.0 (85.0; 162.0)	297	132.0 (103.0; 178.0)	320	102.0 (84.0; 141.0)
**2020**	17	120.0 (86.0; 173.0)	438	95.2 (61.2; 139.0)	685	81.0 (42.0; 115.0)

HC, hypercholesterolemia; HeFH, heterozygous familial hypercholesterolemia; HoFH, homozygous familial hypercholesterolemia; LDL-C, low-density lipoprotein cholesterol; Q, quartile.

Table S12

Reasons for discontinuation of evolocumab in the safety analysis group.

Reason, n (%)	Patients who discontinued* (N=1200)
**Patient preference (excluding AEs)**	373 (31.1)
**Symptom remitted/recovered**	264 (22.0)
**AE**	186 (15.5)
**Hospital transfer**	162 (13.5)
**No visit during the study**	90 (7.5)
**Constant/aggravated symptom**	26 (2.2)
**Other**	16 (1.3)
**Financial reasons**	13 (1.1)
**No visit after the first day of this drug administration**	10 (0.8)
**Change to other PCSK9 inhibitors**	7 (0.6)

AE, adverse event; PCSK9, proprotein convertase subtilisin/kexin type 9.

* Patients with a treatment duration of <104 weeks were withdrawn from treatment. One-hundred nineteen subjects with no reason for discontinuation were excluded from the tabulation; if a patient discontinued treatment more than once, the reason for discontinuation at the time of the last discontinuation of the largest volume was tabulated; if multiple reasons for discontinuation existed at a single discontinuation for the same patient, all relevant reasons were tabulated.

Figure S1. Percentage change (mean±SD) in LDL-C levels from baseline in effectiveness analysis group 2 for patients with (A) HoFH, (B) HeFH, and (C) HC with high risk, and (D) the total population.

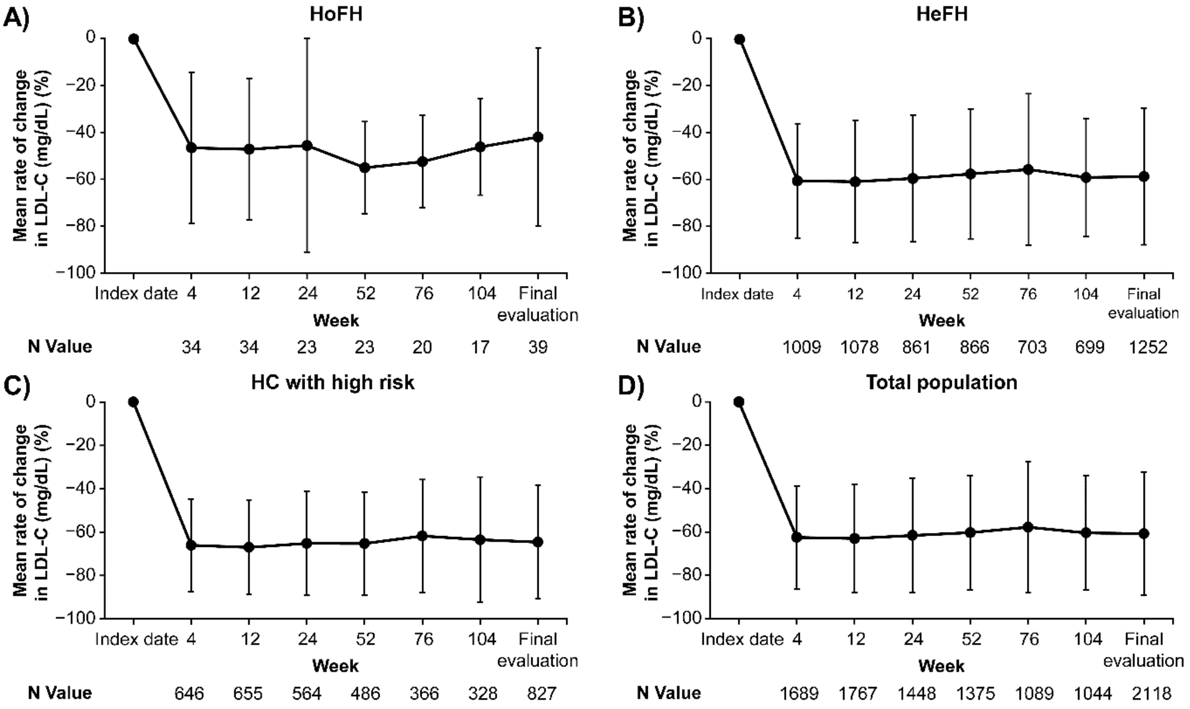

HC, hypercholesterolemia; HeFH, heterozygous familial hypercholesterolemia; HoFH, homozygous familial hypercholesterolemia; LDL-C, low-density lipoprotein cholesterol

N values represent the number of patients who had both LDL-C values at baseline and any time point after the treatment.

Figure S2: Outcome of evolocumab-treated patients (HoFH, HeFH, and HC with high risk) over 104-week follow-up.

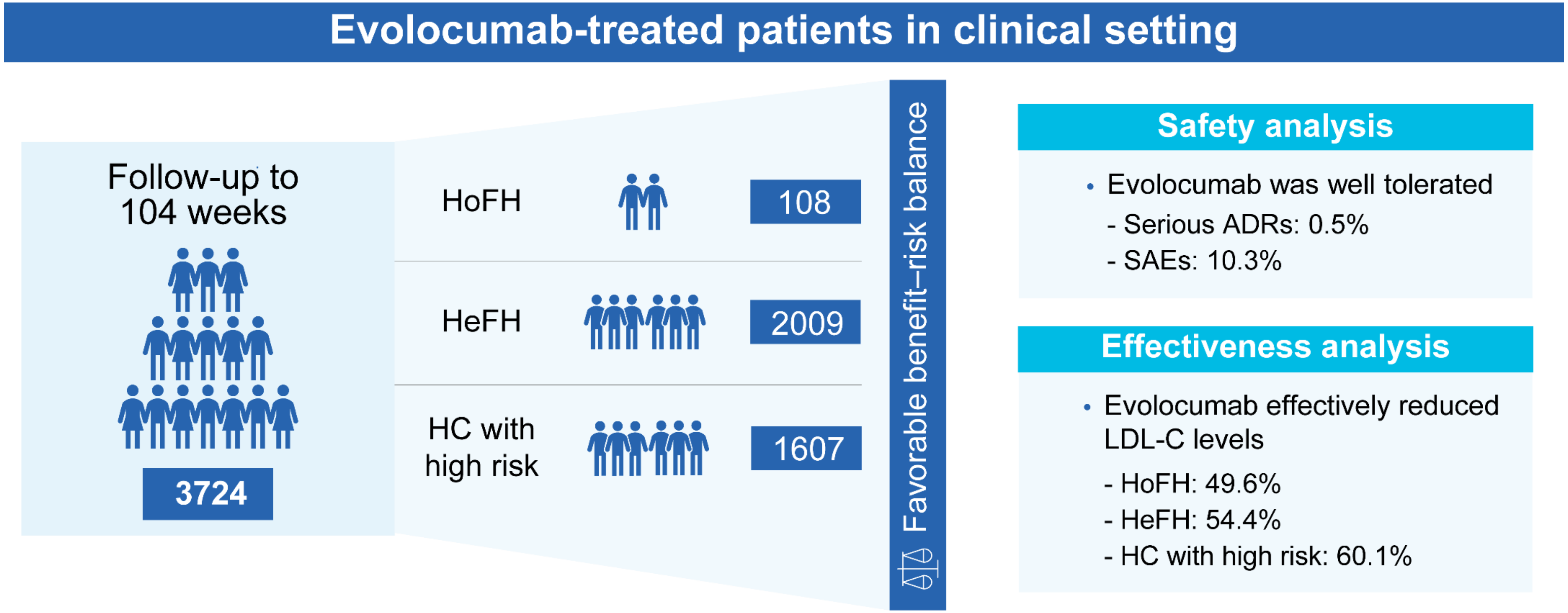

ADR, adverse drug reaction; HC, hypercholesterolemia; HeFH, heterozygous familial hypercholesterolemia; HoFH, homozygous familial hypercholesterolemia; LDL-C, low-density lipoprotein cholesterol; SAE, serious adverse event.
